# Attenuation of near-ultraviolet, visible and near-infrared light in sound and carious human enamel and dentin

**DOI:** 10.1007/s00784-022-04541-7

**Published:** 2022-05-19

**Authors:** Katrin Berghammer, Friederike Litzenburger, Katrin Heck, Karl-Heinz Kunzelmann

**Affiliations:** Department of Conservative Dentistry and Periodontology, LMU Munich, University Hospital, Goethestr. 70, 80336 Munich, Bavaria Germany

**Keywords:** Caries detection, Caries zones, Dentin caries, Enamel caries, Transillumination, Tertiary dentin

## Abstract

**Objectives:**

This in vitro study aimed to investigate the optical attenuation of light at 405, 660 and 780 nm sent through sound and carious human enamel and dentin, including respective individual caries zones, as well as microscopically sound-appearing tissue close to a carious lesion.

**Materials and methods:**

Collimated light transmission through sections of 1000–125-µm thickness was measured and used to calculate the attenuation coefficient (AC). The data were statistically analysed with a MANOVA and Tukey’s HSD. Precise definition of measurement points enabled separate analysis within the microstructure of lesions: the outer and inner halves of enamel (D1, D2), the translucent zone (TZ) within dentin lesions and its adjacent layers, the enamel side of the translucent zone (ESTZ) and the pulpal side of the translucent zone (PSTZ).

**Results:**

The TZ could be distinguished from its adjacent layers and from caries-free dentin at 125 µm. Sound-appearing dentin close to caries lesions significantly differed from caries-free dentin at 125 µm. While sound and carious enamel exhibited a significant difference (*p* < 0.05), this result was not found for D1 and D2 enamel lesions (*p* > 0.05). At 405 nm, no difference was found between sound and carious dentin (*p* > 0.05).

**Conclusions:**

Light optical means enable the distinction between sound and carious tissue and to identify the microstructure of dentin caries partially as well as the presence of tertiary dentin formation. Information on sample thickness is indispensable when interpreting the AC.

**Clinical relevance:**

Non-ionising light sources may be suitable to detect lesion progression and tertiary dentin.

## Introduction

Light optical diagnostic methods do not require X-rays and are considered a promising alternative to conventional caries diagnostic methods. The established techniques, mainly represented by visual/tactile or radiographic methods, have imperfections in diagnostic accuracy in regard to assessing the presence or severity of a carious lesion [1–4]. A variety of commercially available non-ionising light optical diagnostic systems have been introduced to the dental market in recent decades. Detection methods using monochromatic light sources at different wavelengths include quantitative light-induced fluorescence measurement at 405 nm (QLF), laser fluorescence measurement at 655 nm (LF), as in DIAGNOdent (KaVo, Biberach, Germany), and near-infrared transillumination at 780 nm (NIRT), as in DIAGNOcam (KaVo, Biberach, Germany). These techniques have been described as suitable adjunct options for visual examination to detect early carious lesions and they possess a diagnostic performance similar or even superior to that of bitewing radiography [5–7]. However, to date, none of these methods has been able to seriously compete with bitewing radiography as the established auxiliary method for caries diagnosis, because they cannot reliably assess lesion depth [7–9]. Bitewing radiographs directly depict carious processes in relation to the enamel-dentin junction (EDJ) and to the pulp with high specificity, which is their major and unique advantage. This enables dentists to evaluate the severity of carious lesions. Knowledge of the actual depth of a non-cavitated lesion provides valuable information for deciding whether non-invasive, minimally invasive or invasive therapy is adequate. Hence, this knowledge is relevant not only to merely detecting a carious lesion in dentin or enamel but also to diagnosing its severity to derive a therapy and prognosis [10].

When examining the microstructure of a carious lesion, enamel lesions can be subdivided into an outer half (D1) and an inner half (D2) [11]. In dentin lesions, the translucent zone (TZ; zone of dentinal sclerosis) can be clearly defined when present, which allows for characterisation of two further regions: the enamel side of the translucent zone (ESTZ) and the pulpal side of the translucent zone (PSTZ). Depending on lesion depth, the ESTZ may comprise the zone of necrosis, the zone of penetration, the zone of demineralization and dead tracts. The PSTZ may comprise a layer of sound dentin and tertiary dentin [12]. The optical properties of these different zones of a dentin lesion are not well known. The propagation of light within sound and decayed dental hard tissues is a complex process due to their heterogeneous microstructure [13]. The attenuation of light is mainly defined by the scattering properties of the materials and decreases with increasing wavelength, while the absorption caused by intracrystalline or intratubular fluids has a subordinate impact on the propagation of light [14, 15]. The apatite crystals within the organised crystalline structure of sound enamel scatter light weakly, which results in low attenuation of light and high transparency. Demineralisation of enamel is associated with the growth of pores functioning as additional scattering centres. Previous studies with wavelengths ranging from 543 to 1310 nm demonstrated that sound enamel exhibits a significantly lower attenuation than decayed enamel [15–17]. In dentin with its higher organic content, scattering is mainly caused by fluid-filled tubules [18]. Sound and carious dentin attenuate light to a greater extent than sound enamel [16]. However, the current literature provides little information regarding the contrast between sound and carious dentin. Hoffmann et al. investigated the optical properties of sound and carious dental hard tissues for a wavelength range of 532–780 nm using goniometric measurements and confirmed that—in contrast to enamel tissues—the attenuation of light was stronger in sound dentin than in decayed dentin [18, 19].

The aim of this study was to analyse the attenuation coefficients of sound dental hard tissues, sound-appearing tissue in close proximity to a carious lesion and zones within carious lesions using near-ultraviolet, visible and near-infrared light. Therefore, transillumination measurements were performed on tooth samples of decreasing thickness ranging from 1000 to 125 µm. Referring to current commercially available diagnostic devices QLF, DIAGNOdent and DIAGNOcam, the measurements were performed using light at 405, 660 and 780 nm.

The following working hypotheses were formulated: The attenuation coefficients (ACs) of sound and carious dental hard tissues differ significantly. Sound-appearing dentin and enamel in proximity to carious lesions exhibit the same optical properties as enamel and dentin of caries-free samples. The ACs measured in different zones of enamel and dentin carious lesions show significant differences within the respective tissue. The sample thickness has no effect on the AC throughout the examined wavelength spectrum.

## Materials and methods

### Sample preparation

Forty extracted human posterior teeth of anonymous patients were cleaned and stored in Ringer’s solution containing 2% sodium azide at 4 °C. The teeth were visually examined concerning signs of carious defects. Twenty teeth were free of caries and twenty teeth exhibited (mainly occlusal) carious lesions of varying extent with and without microcavitation. The Ethics Committee of the Medical Faculty of the Ludwig-Maximilians University of Munich approved the use of unidentified pooled human teeth (approval no. 488–15 UE).

The teeth were cut in the labio-lingual direction (IsoMet Low Speed Saw 11–1280-250; Buehler, Lake Bluff, IL, USA) and manually polished on one side (LECO grinding & polishing SS-200; LECO Corporation, Saint Joseph, MI, USA) with SiC grinding papers of 600–2500 grit and diamond polishing spray. After removing the pulp and washing the sample with water, it was air-dried and its polished side was fixed to a specimen slide (41500, EXAKT Apparatebau, Norderstedt, Germany; and Technovit 7210 VLC Präzisionskleber, Heraeus Kulzer GmbH, Hanau, Germany). Next, the other side of the specimen was ground and polished by an automated grinding system (Mikro-Schleifsystem EXAKT 400 CS; EXAKT Apparatebau, Norderstedt, Germany) to reach the desired initial sample thickness of 1000 µm, which was validated using a digital micrometre (MarCator 1086 W, Mahr GmbH, Esslingen, Germany). Carious teeth were cut in the area of a lesion and visually examined. Only tooth slices that exhibited a carious area that extended throughout the whole thickness of the specimen were processed further. All samples were washed with water to remove abrasive particles from the grinding process and stored in tap water at 4 °C. The thickness of all specimens was gradually mechanically reduced to 500 µm, 250 µm and 125 µm after transillumination measurements were performed for each thickness.

### Sample measurement points

Within caries-free samples, two measurement points each in sound enamel (SE) and sound dentin (SD) in the area of the cusps were determined, resulting in a total of 40 readings per tissue. Within carious tissue, a varying number of measurement points at intervals of approximately 500 µm were determined. Measurement points in carious enamel (CE) were grouped according to their position in the outer half (D1) and inner half (D2) of the lesion. Measurement points in dentin caries (CD) were grouped in terms of their position relative to the TZ.

**Fig. 1:**
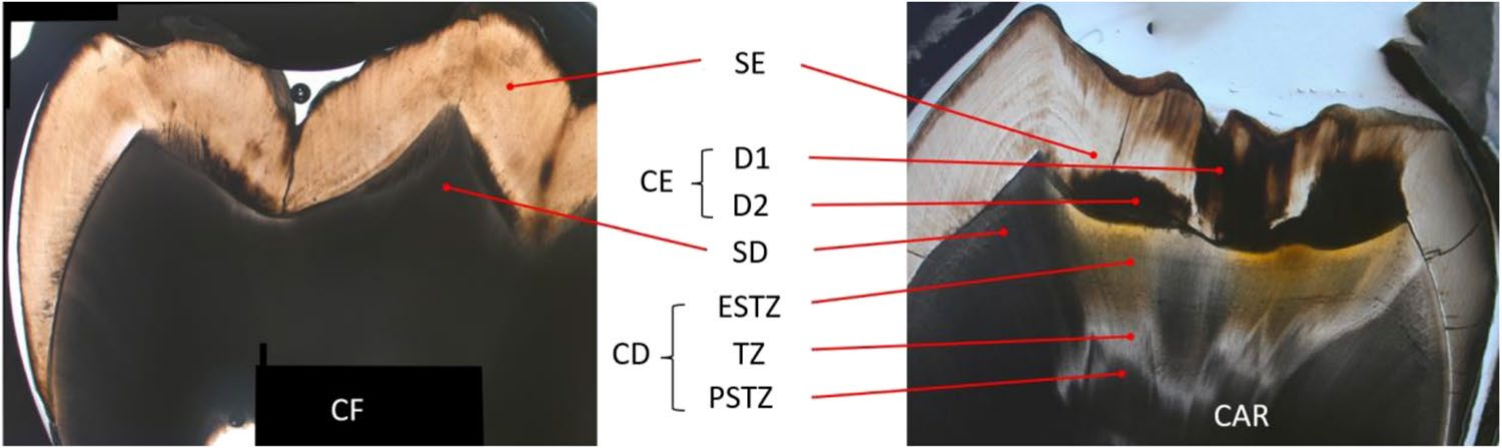
Measurement points in a caries-free (CF) sample and a carious (CAR) sample. SE, sound enamel; SD, sound dentin; CE, carious enamel; D1, enamel caries outer half; D2, enamel caries inner half; CD, carious dentin; PSTZ, pulpal side of the translucent zone; TZ, translucent zone; ESTZ, enamel side of the translucent zone

They were located either within the TZ, on the ESTZ or on the PSTZ. For samples affected only by superficial dentin caries, the TZ was not visible, which is why in this case, all measurement points were classed as ESTZ. Additionally, one measurement point each was set in visually sound-appearing enamel and dentin (SE, SD) in proximity to an enamel carious lesion and a dentin carious lesion to enable a comparison between sound tissues of caries-free and carious samples and to evaluate whether the optical properties of dental hard tissues changed because of the pulp’s reaction to the carious stimulus. The exact position of each measurement point was noted to be accurate to 1/10 mm and thereby reproducible throughout the investigation. Figure 1 illustrates the positions of the measurement points with reference to images of a caries-free sample and a carious sample.

### Measurement of the light intensity

A microscope (Axioskop 2 MAT, Carl Zeiss, Göttingen, Germany) with a magnification lens (Zeiss EC Epiplan-NEOFLUAR, 20 × /0.5 HD DIC, Carl Zeiss, Göttingen, Göttingen, Germany) and a spectrometer (Fibre Optic Spectrometer SD2000, Ocean Optics, Dunedin, FL, USA) were utilised for the transillumination measurements. Three different light-emitting diodes (LEDs) operating in continuous-wave mode at 405, 660 and 780 nm (M405L2/M660L3/M780L2, Thorlabs Inc., Newton, NJ, USA) served as light sources. They were powered by an external power supply (High Power LED Driver DC2100, Thorlabs Inc., Newton, NJ, USA) at 28 mA, 20 mA and 340 mA respectively. Before measuring the samples, the blank value “*I*_0_” was obtained to account for the attenuation of light caused by the specimen slide. The samples were placed on the microscope table, focussed and visually inspected using a halogen lamp (HLX 64623, 100 W, 12 V; OSRAM GmbH, Augsburg, Germany) and a 20 × magnification lens to choose respectively examine the measuring points lying within the respective tissue before the first measurement and after each grinding process. It was made sure that only the desired tissue filled the whole field of view. The specimens were covered with a thin layer of tap water during the examination to avoid corruption of measurement results due to desiccation. The intensities for the abovementioned measurement points were recorded for all light sources at sample thicknesses of 1000, 500, 250 and 125 µm. The collimated signal (*I*) at the detector was compared to the initial intensity (*I*_0_) of the beam. These raw data were processed by an analogue–digital converter (External USB A/D Converter: ADC 1000-USB; Ocean Optics, Dunedin, FL, USA) and SpectraSuite software (Spectrometer Operating Software, Ocean Optics, Dunedin, FL, USA).

### Statistics

The attenuation coefficient (AC) was calculated using the ratio of the intensity at the detector (*I*) to the initial intensity (*I*_0_) and the sample thickness (*t*):

1 $$\mathrm{AC} =\frac{\mathrm{ln}\left(I0\right)-\mathrm{ln}\left(I\right)}{t}$$

Statistical analysis was performed using SPSS (IBM SPSS Statistics for Windows, v.25.0, Armonk, NY, USA) and the computing environment R (R Development Core Team, 2019) [20]. Mean values and standard deviations of the AC were calculated. The data were statistically analysed with a MANOVA and post hoc testing was performed using Tukey’s HSD. A *p* value < 0.05 was considered significant.

## Results

**Fig. 2:**
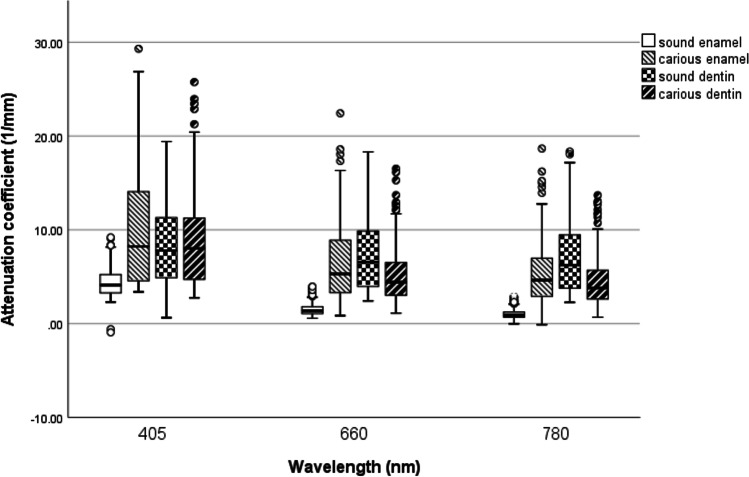
Box plot of the attenuation coefficients of sound and carious enamel and dentin at 405 nm, 660 nm and 780 nm

**Fig. 3:**
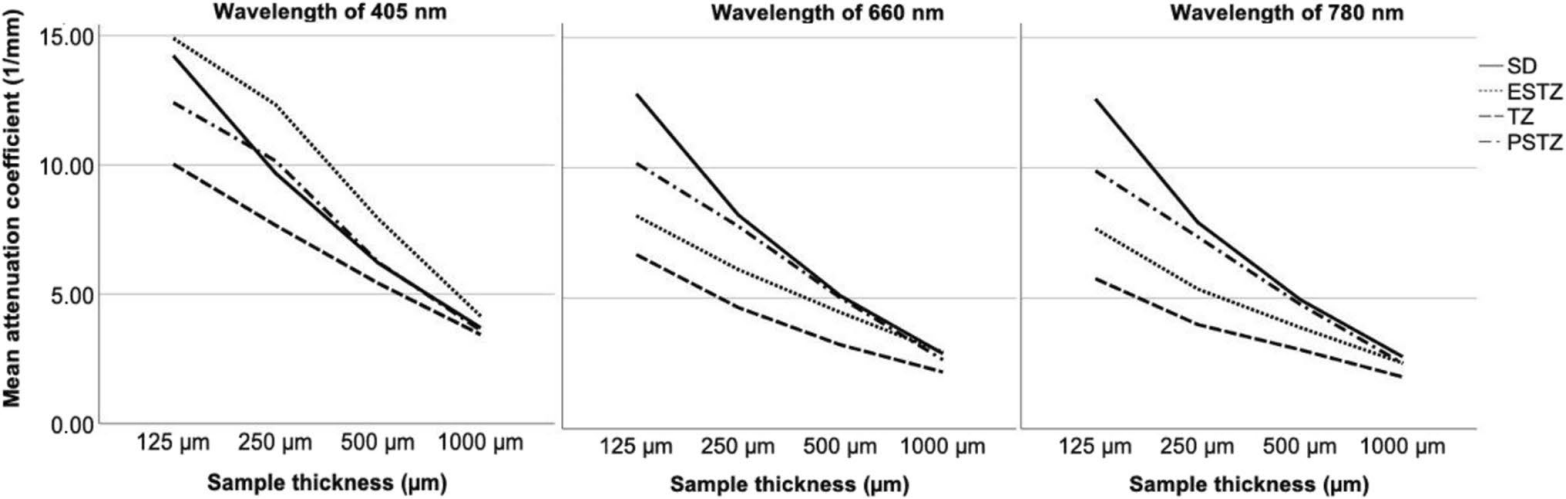
Mean attenuation coefficients of sound dentin (SD) and different zones of carious dentin (PSTZ, pulpal side of the translucent zone; TZ, translucent zone; ESTZ, enamel side of the translucent zone) in relation to the sample thickness and wavelength

Figure 2 shows a box plot of the ACs of enamel and dentin at 405 nm, 660 nm and 780 nm. A statistical explanation for the wide range of values is that the data incorporate the ACs for all the sample thicknesses. At 405 nm carious enamel showed the highest AC, while at 660 nm and 780 nm, the highest AC could be observed for sound dentin (Fig. 2). Generally, the ACs of the different tissues approached each other with increasing sample thickness (Fig. 3; Table 1). The ACs of all tissue types were higher at lower wavelengths and increased with decreasing sample thickness. The ACs exhibited a high variation with changing sample thickness.

**Table 1:** Attenuation coefficients (1/mm) for different measurement points in sound and carious sections of posterior teeth depending on wavelength (405 nm, 660 nm, 780 nm) and sample thickness (1000–125 µm), with the standard deviation in parentheses

	Enamel						Dentin					
	Sound		Carious				Sound	Carious				
	SE		SE	CE	CE D1	CE D2	SD	SD	CD	CD ESTZ	CD TZ	CD PSTZ
405 nm												
1000 µm	2.84 (0.87)	3.07 (0.30)		4.11 (0.38)	4.04 (0.46)	4.14 (0.34)	3.55 (0.68)	3.70 (0.33)	4.00 (0.46)	4.15 (0.32)	3.44 (0.58)	3.60 (0.21)
500 µm	3.92 (0.54)	4.17 (1.37)		7.75 (1.09)	7.75 (0.97)	7.75 (1.18)	6.24 0.49)	6.20 (0.62)	7.30 (1.62)	7.92 (1.24)	5.42 (1.53)	6.24 (0.64)
250 µm	4.86 (0.85)	4.88 (1.11)		12.26 (4.07)	13.11 (3.95)	11.82 (4.15)	10.13 (0.95)	9.67 (0.89)	10.94 (3.31)	12.32 (2.83)	7.66 (1.90)	10.15 (3.19)
125 µm	5.67 (1.26)	6.11 (2.27)		16.44 (7.92)	18.31 (7.91)	15.47 (7.93)	15.48 (2.10)	14.22 (2.04)	13.34 (5.28)	14.90 (5.27)	10.03 (4.36)	12.41 (3.22)
660 nm												
1000 µm	1.14 (0.25)	1.29 (0.51)		3.24 (0.51)	3.35 (0.47)	3.18 (0.53)	2.93 (0.26)	2.88 (0.31)	2.80 (2.70)	2.94 (0.66)	2.17 (0.47)	2.65 (0.15)
500 µm	1.32 (0.36)	1.34 (0.40)		5.43 (1.51)	5.35 (1.80)	5.46 (1.39)	5.25 (0.43)	5.09 (0.57)	4.25 (1.16)	4.46 (1.14)	3.22 (0.72)	5.01 (0.65)
250 µm	1.65 (0.73)	1.72 (0.57)		7.74 (3.62)	8.65 (3.88)	7.26 (3.48)	8.62 (0.96)	8.19 (0.85)	5.89 (2.06)	6.10 (1.95)	4.65 (1.61)	7.76 (2.28)
125 µm	2.19 (1.53)	2.47 (1.46)		9.15 (5.70)	10.35 (5.52)	8.52 (5.81)	14.18 (2.42)	12.84 (1.42)	7.94 (4.26)	8.17 (4.36)	6.68 (4.26)	10.18 (2.71)
780 nm												
1000 µm	0.77 (0.19)	0.92 (0.53)		2.86 (0.43)	2.97 (0.40)	2.80 (0.45)	2.82 (0.25)	2.76 (0.32)	2.43 (0.48)	2.52 (0.47)	1.99 (0.39)	2.50 (0.15)
500 µm	0.92 (0.35)	0.92 (0.34)		4.78 (1.34)	4.76 (1.55)	4.80 (1.26)	5.16 (0.47)	4.95 (0.56)	3.78 (1.00)	3.88 (0.98)	3.03 (0.66)	4.77 (0.78)
250 µm	1.05 (0.52)	1.17 (0.52)		6.54 (3.30)	7.48 (3.49)	6.05 (3.16)	8.30 (0.92)	7.91 (0.82)	5.19 (2.00)	5.36 (1.87)	4.00 (1.65)	7.36 (1.90)
125 µm	1.37 (0.83)	1.67 (1.04)		7.64 (5.08)	8.50 (5.01)	7.20 (5.17)	14.12 (2.22)	12.65 (1.72)	7.36 (4.19)	7.67 (4.20)	5.76 (4.12)	9.89 (3.08)

*SE*, sound enamel; *SD*, sound dentin; *CE*, carious enamel; *D1*, enamel caries outer half; *D2*, enamel caries inner half; *CD*, carious dentin; *PSTZ*, pulpal side of the translucent zone; *TZ*, translucent zone; *ESTZ*, enamel side of the translucent zone

Sound enamel significantly exhibited the lowest AC for all categories (*p* < 0.001) (Table 1). The contrast between sound and decayed enamel was most significant within the examined wavelength range for thicknesses of 125 to 500 µm (*p* < 0.05) but not significant for a sample thickness of 1000 µm (*p* > 0.05). For the zones of enamel caries D1 and D2, no significant difference could be found within the examined wavelength range at the same sample thickness (*p* > 0.05).

Independent of the sample thickness, no significant difference could be found between the ACs of sound and decayed dentin at 405 nm (*p* > 0.05). In contrast, a statistically significant difference between sound and carious dentin was found for sample thicknesses of 125 µm and 250 µm at wavelengths of 660 nm and 780 nm (*p* < 0.001). For sample thicknesses ≥ 500 µm, no significant difference was found (*p* > 0.05). Comparing the zones of carious dentin, the lowest AC was found for the TZ for all the categories (Fig. 3; Table 1). For 660 nm and 780 nm, the highest AC was found within the PSTZ of carious dentin up to a thickness of 500 µm (Fig. 3; Table 1). With increasing wavelength from 660 to 780 nm and decreasing sample thickness, the difference between the dentin carious zones became more evident; however, the difference was not significant at any of the sample thicknesses (*p* > 0.05). In contrast, at 405 nm, the ACs revealed a different trend. Here, the ESTZ represented the zone with the highest AC, which was even higher than the AC of sound dentin regardless of sample thickness (*p* > 0.05) (Fig. 3; Table 1). A statistically significant difference was found between the TZ and ESTZ up to a thickness of 250 µm (*p* < 0.001).

Furthermore, a significant difference was found between sound-appearing dentin close to a carious lesion and caries-free dentin at a sample thickness of 125 µm for all wavelengths, but this difference ceased to be significant at higher thicknesses. Sound-appearing enamel close to an enamel carious lesion showed no significant difference.

## Discussion

The focus of this study was to investigate the attenuation properties of sound and carious enamel and dentin, including their ultrastructure, using light ranging between 405 and 780 nm. Monochromatic and incoherent light sources operating at 405 nm, 660 nm and 780 nm were selected to represent some of the most common light optical diagnostic methods available, namely, QLF, LF as in DIAGNOdent and NIRT as in DIAGNOcam, and to provide important physical data which have not been reported by the literature and the manufacturers yet. Since all data were acquired in one study, under the exact same experimental conditions, this enables a direct comparison of the performance of light sources operating at different wavelengths. Transillumination measurements of sound and carious specimens at gradually decreasing sample thicknesses ranging between 1000 and 125 µm were performed, followed by calculation of the AC. One of our initially formulated hypotheses, that the ACs of the individual zones of carious tissue and the ACs of sound and carious tissue differ significantly for all investigated wavelengths, was only partially confirmed by the results of this study. Regarding carious enamel, no significant differences were found between D1 lesions and D2 lesions (Table 1). The outer layers of enamel lesions—the surface layer and the body of lesion—show a high degree of demineralisation and high pore volume during lesion formation, whereas the dark zone and translucent zone of enamel caries show less demineralisation [12]. This is why we presumed that the attenuation by D1 lesions would be stronger than by D2 lesions. In previous research, Darling et al. compared sound and carious enamel according to high-resolution microradiographic images to precisely characterise their scattering properties. The scattering of enamel lesions was found to exponentially grow with the level of demineralisation and reached its maximum after 10–15% mineral loss [13]. In this context, we presume that all lesions examined in the present study have exceeded this critical point of mineral loss, resulting in like scattering by the porous tissue, and consequently, no difference between D1 and D2 lesions was detectable by the measurement methods applied here. However, this cannot be proved since no mineral content measurements were performed.

The contrast between the TZ of a dentin lesion and sound dentin was visible to the naked eye. In this translucent portion, the odontoblasts had deposited more minerals inside the dentinal tubules, which protects the pulp from bacterial toxins. In the underlying PSTZ, these intratubular crystals had increased in size and number, as reported by Ogawa et al. [21]. The subdivision in the three layers ESTZ, TZ and PSTZ was chosen, because they could be easily identified and reproduced, whereas the microscopic differentiation of single histologic zones is complex and there is no clear cutoff line between them, but some zones show a merging transition [12, 22]. Precise and accurate selection of measurement points, which underlines the high quality of the examination, allowed the transillumination measurements of the different caries layers to be compared with those of nearby sound-appearing dentin. To our knowledge, the attenuation characteristics of enamel and dentin have not been studied in such detail before. Figure 3 shows similar ACs for measurements in sound and carious dentin at different wavelengths, depending on the sample thickness. Although the ACs decreased and converged with increasing sample thickness at all wavelengths, the attenuation of light in the ultraviolet wavelength range (405 nm) appeared to be fundamentally different from that at longer wavelengths. The highest AC, at any sample thickness, was found for the ESTZ at a wavelength of 405 nm, while at higher wavelengths, the highest AC was observed for sound dentin and the PSTZ. This result seems reasonable since the dentin tubules are the main light scatterers and their number and density rise with increasing proximity to the pulp. The difference between the ESTZ and TZ at a wavelength of 405 nm was significant for sample thicknesses of 125 to 250 µm but disappeared at higher sample thicknesses. Our results revealed that the AC for the TZ reached lower values than those for the adjacent caries layers (ESTZ, PSTZ) and caries-free dentin throughout all examined categories. This was especially noticeable for thin samples, but except at 405 nm, no statistical significance was found. The higher transmission of light within the TZ can be explained by the change in tubule structure. The obliteration of tubules causes two boundary layers for light refraction and scattering to vanish [23]. For ultraviolet light and a sample thickness of 1000 µm, the effect was less evident. For the dentin of caries-free samples, the refractive index between the tubules formed by the collagen matrix and the intratubular fluid was predominantly uniform, so light at 405–780 nm was almost completely and homogeneously refracted. Attenuation was higher for the PSTZ than for the TZ at wavelengths of 660 nm and 780 nm.

Considering all parameters, the results of the measurements at 660 nm and 780 nm showed the same trends (Fig. 3). Our observations and the results of the previous publications mentioned earlier revealed that only at higher wavelengths can different layers of a dentin lesion be distinguished from one another and from caries-free dentin. This is a partial disconfirmation of our first hypothesis.

The ability to distinguish between sound and carious tissue, especially within dentin close to the pulp, is an essential requirement for the development of future caries diagnostic systems, where artificial intelligence may be able to contribute to automated and objectified diagnostics and ultimately therapy [24–26].

Furthermore, we hypothesised that sample thickness would have no impact on the AC regardless of examination wavelength. This assumption must be rejected, as decreasing the thickness of sound and diseased samples proved to be associated with an increase in AC. The AC varied strongly between sample thicknesses of 125 and 1000 µm. A normalisation of the AC for dental material with a general single value valid for all sample thicknesses (calculation analogous to the Lambert–Beer law), as used in previous research, cannot be recommended [18].

Our measurements were carried out in a laboratory setup that did not completely mimic the clinical situation, which must be associated with the outcomes of this study. We chose to cut the teeth in the labio-lingual direction and to work with mesio-distal transillumination of the samples to obtain a large area of dentin and enamel that would persist throughout the thickness reduction process. The longitudinal cutting direction was chosen because it enabled us to inspect all histologic layers of carious lesions at once. Since enamel and dentin possess anisotropic qualities, they exhibit varying optical properties depending on the incidence of light. For enamel, the lowest attenuation is observed with light incident from the labio-lingual direction, while for dentin, this is true for light incident from the mesio-distal direction. These anisotropic properties are much less pronounced for enamel than for dentin [27]. The orientation of the light beam and cutting direction have been reported to influence light propagation in dentin due to the complex non-homogeneous structure of the matrix [28–30]. Ultimately, the influence of the cutting direction on the diagnostic outcome of this investigation was considered negligible. All measurement points were chosen by microscopic examination using a 20 × magnification lens. The overall spot size of the magnification lens reaches ~ 1 mm, which means that when measuring several points within a carious lesion at a distance of ~ 500 µm, it is possible that there were overlaps at the outer border of the examined area. The experiment setup of this study was based on simple transillumination measurements and did not reproduce the complex mechanisms of the commercially available caries diagnosis tools mentioned above, which is why the results cannot be considered equivalent to those of clinical studies using the actual devices. This study does however provide the physical basic information.

Figure 2 exhibits a wide range of values, especially for measurements in sound and carious dentin. One reason for this is that these comparisons do not consider the different sample thicknesses but are calculated according to the mean values for all thicknesses. Another explanation for this wide range of values is the natural anatomical variation of biological tissues, which often leads to larger variations in measurements compared to non-biological material. The potentially high degree of measurement variation suggests setting a reference value in caries-free dental tissue for each patient examined with light optical diagnostic systems. The influence of varying the wavelength from 405 to 780 nm on the optical properties of enamel and dentin demonstrated confirmatory facts: the dental hard tissues appeared more transparent with increasing wavelength, and their ACs decreased accordingly. Monochromatic light at 405 nm exhibited potential to discriminate between sound and diseased enamel as well as between enamel and dentin, while discrimination between sound and decayed dentin tissues was impossible. At 660 nm and particularly 780 nm, a constant difference could be measured between sound and carious enamel and dentin and even—which is novel information—between the different zones of a carious lesion in dentin. A promising future research project would be to analyse the potential of monochromatic light at higher wavelength ranges between 800 and 1600 nm for stronger discrimination of the different zones of dentin caries. Since higher wavelengths cause less light scattering, better light penetration and potentially better differentiation of tissue types are possible. For said wavelength range, literature provides attenuation coefficients between ~ 260 and 20 cm^−1^. Chan et al. found a wavelength range of 1300–1400 nm to be most preferable for transillumination measurements of dentin [16, 18].

During transillumination measurements, the specimens were covered with water but not fully immersed, e.g. in a container filled with liquid. We omitted the use of an index-matching fluid in this context, as this would lead to optical obscuration of the lesions [17]. The use of water containing sodium azide could also have a difficult-to-calculate effect on the results of our measurements, which is why we stored the samples in pure tap water between the measurements. All samples were washed after each grinding process. The smear layer was not removed, since it has only a minor effect on light attenuation in dentin and because sonicating the samples before each measurement would increase the risk of fractures, especially for thinner samples [31]. These procedures could have confounded the results of the comparison between caries-free dentin and sound-appearing dentin found in proximity to carious lesions. At a sample thickness of 125 µm, the dentin located close to carious dentin exhibited significantly different ACs compared to the sound dentin of caries-free samples. This fact has not yet been reported by any previous research. This phenomenon might be due to the formation of tertiary dentin caused by the pulp’s reaction to the carious stimulus. Since the dentinal tubules are then filled, the refractive index becomes similar to that of the surrounding dentin, which results in less light scattering. Future analyses at higher wavelength ranges above 1000 nm could shed light on this in more detail. However, it can be stated that based on these measurements, a potentially sound-appearing surface of a carious tooth should not be used for calibration measurements for light optical methods. Instead, a caries-free tooth should be used to set a reference value. Furthermore, the results of this study indicate that the diagnostic potential of light optical diagnostic methods is not yet exhausted, as characterisation of dentin lesion severity is theoretically possible. In the future, it is a scientific challenge to extrapolate these findings to teeth that are preserved in their entirety.

## Conclusion

Carious enamel could be distinguished from sound enamel at all wavelengths tested. The discrimination between sound and carious dentin was possible at 660 nm and 780 nm. Monochromatic light ranging from 405 to 780 nm was not suitable for discriminating between D1 and D2 enamel lesions. In tooth sections of 125 µm thickness, it showed the potential to reveal the difference between the individual layers of dentin caries. The TZ could be discriminated from its adjacent layers, the ESTZ and PSTZ, as well as from sound dentin. With the difference between sound-appearing dentin close to carious lesions and caries-free dentin, the presence of tertiary dentin formation could be noted by light optical means. A potentially sound-appearing surface of a carious tooth should not be used for calibration measurements for light optical methods since this may lead to imprecise measurements. Reference values should only be set in caries-free teeth. Generally, the AC of all tissue types was higher at lower wavelengths and increased with decreasing sample thickness. The AC results changed with sample thickness and therefore cannot be interpreted independently of this information. It should be considered to integrate a mechanism for the registration of sample thickness in future light optical diagnostic systems that interpret absolute measurement values.

## Data Availability

The study materials and data can be requested in writing from the corresponding author.
